# Sense of Place, Fast and Slow: The Potential Contributions of Affordance Theory to Sense of Place

**DOI:** 10.3389/fpsyg.2017.01674

**Published:** 2017-09-29

**Authors:** Christopher M. Raymond, Marketta Kyttä, Richard Stedman

**Affiliations:** ^1^Department of Landscape Architecture, Planning and Management, Swedish University of Agricultural Sciences, Uppsala, Sweden; ^2^Department of Built Environment, Aalto University, Espoo, Finland; ^3^Department of Natural Resources, Cornell University, Ithaca, NY, United States

**Keywords:** ecological psychology, place meanings, human–environment relationships, place attachment, embodied cognition, affordances, dual-process models

## Abstract

Over the past 40 years, the sense of place concept has been well-established across a range of applications and settings; however, most theoretical developments have “privileged the slow.” Evidence suggests that place attachments and place meanings are slow to evolve, sometimes not matching material or social reality (lag effects), and also tending to inhibit change. Here, we present some key blind spots in sense of place scholarship and then suggest how a reconsideration of sense of place as “fast” and “slow” could fill them. By this, we mean how direct and immediate perception–action processes presented in affordance theory (resulting in immediately perceived place meanings) can complement slower forms of social construction presented in sense of place scholarship. Key blind spots are that sense of place scholarship: (1) rarely accounts for sensory or immediately perceived meanings; (2) pays little attention to how place meanings are the joint product of attributes of environmental features and the attributes of the individual; and (3) assumes that the relationship between place attachment and behavior is linear and not constituted in dynamic relations among mind, culture, and environment. We show how these blind spots can begin to be addressed by reviewing key insights from affordance theory, and through the presentation of applied examples. We discuss future empirical research directions in terms of: (1) how sense of place is both perceived and socially constructed; (2) whether perceived and socially constructed dimensions of place can relate to one another when perceived meanings become unsituated; and (3) how place attachment may change over different stages of the life course based upon dynamic relationships between processes of perception–action and social construction. We conclude with insights into how processes of perception–action and social construction could be included in the design and management of urban landscapes.

## Introduction

Interest in sense of place has grown rapidly in recent years, with the concept extended from leisure and recreation to a wide range of applications and settings. The concept broadly describes human connection to places, including place attachment and place meaning (Stedman, 2003; Farnum et al., 2005; Smaldone et al., 2005). Place attachment refers to the emotional bonds between an individual and a geographic locale, or how strongly a person is connected to a place (Low and Altman, 1992; Jorgensen and Stedman, 2001; Raymond et al., 2010), whereas place meaning is the descriptive, symbolic meaning that people ascribe to a place (Smaldone et al., 2008; Stedman, 2008, 2016).

**FIGURE 1:**
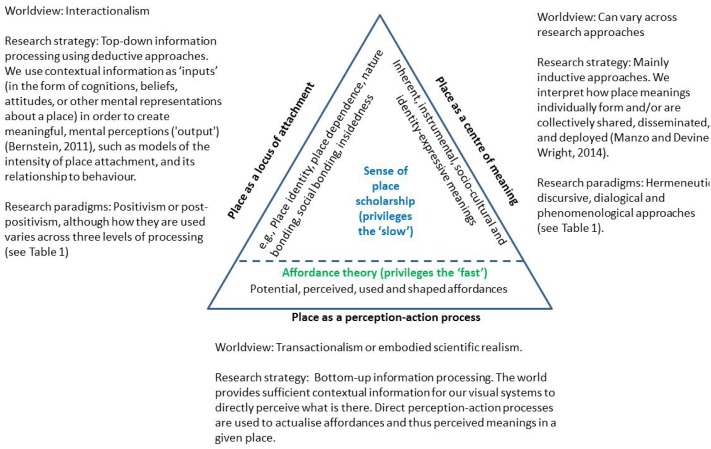
Three perspectives of place considered in this paper.

Historically, sense of place research, including emphases on both meaning and attachment (**Figure 1**), has “privileged the slow.” We see this in the “conservativism” of place critique(s) which views place meanings as slow to evolve, sometimes not matching material or social reality (lag effects), and also place meanings as tending to inhibit change (the maladaptive nature of place meanings) (Marshall et al., 2012; Stedman, 2016; Masterson et al., 2017). It is also generally accepted that place attachment endures over time (Giuliani, 2003; Lewicka, 2011), and slowly changes in intensity or structure with one’s length of residence in a given place (Hammitt et al., 2004; Brown and Raymond, 2007), or waxing and waning over the course of one’s connection to a place (Smaldone et al., 2008). Its stability can be affected by life stage (Elder et al., 1996), by economic, social, political, and other external disruptions to a place (Brown and Perkins, 1992; Feldman, 1996; Devine-Wright, 2009), and across short-term and long-term residents (Kaltenborn and Williams, 2002; Stedman, 2006). Despite these limited engagements of variability, the general tendency in research has been to emphasize the steady, “slow” development of strong attachments and stable meanings.

Within sense of place scholarship, Williams (2014a) identifies two branches of enquiry: “place as a locus of attachment” and place as a “center of meaning” (**Figure 1**). The former refers to an operational construct designed to measure the intensity or strength of a bond to a geographic locale. Scholars rarely acknowledge that this branch of enquiry is based on an interactionist worldview in psychology (Altman and Rogoff, 1987). In this worldview: (1) reality comes divided into subjects and objects in that aspects of the environment are seen as independent of the properties of human minds or bodies (Altman and Rogoff, 1987; Lakoff and Johnson, 1999); (2) the individual is driven by factors located outside in the surroundings, including biological determinants; and (3) the bases for change in the state of the individual are the impacts stemming from entities and conditions in the surroundings, as well pushes from within the individual (Heft, 2013). Researchers commonly employ top-down information processing strategies using deductive approaches. Contextual information is used as “inputs” (in the forms of cognitions, beliefs, attitudes, or other mental representations about a place, see Williams, 2014a) in order to create meaningful mental perceptions (“output”) (Bernstein, 2010).

In contrast, “place as a center of meaning” refers to the broader processes of meaning-making, and how to characterize experience, meaning, and relationships to places in more experiential qualitative terms (Williams, 2014a). Emphasis is placed on an interpretative approach to cognition (Bruner, 1990), with hermeneutic, discursive, dialogical, or phenomenological research paradigms used to interpret how place meanings form, or are collectively shared, disseminated and deployed (see **Table 1** and section “Sense of Place Scholarship Rarely Accounts for Sensory or Immediately Perceived Meanings” for further explanation).

**Table 1:** An overview of the differing approaches to sense of place and affordance scholarship.

	Foci of place	Research strategy	Different research paradigms or approaches used to understand place			
Sense of place scholarship (privileges the “slow”)	Place as a center of meaning (focus on place meaning)	*Inductive*. We interpret how place meanings individually form and/or are collectively shared, disseminated, and deployed	*Hermeneutic approach*. Meaning is generated in the individual subjective mind through the interpretation of texts. Place has to be interpreted in order to reveal a deeper meaning. Landscape legibility is therefore a key to understanding place meaning (Drenthen, 2011).	*Discursive approach*. Place meanings are treated as a social practice that cannot be understood outside of interactional, cultural, and institutional contexts in which they emerge. They are formed through everyday language use and social practice and have significant rhetorical relevance (Di Masso et al., 2014).	*Dialogical approach*. “Meaning arises out of the relationship between an act and those trying to understand it – it is the product of an interaction of two subjects” (Fay, 1996, p. 142). Dialogical approaches highlight the central role of actions and practices in producing place meaning (West, 2016).	*Phenomenological approach*. A holistic approach to cognition whereby individual or group actions, experiences, intentions, and meanings are drawn together spatially (Relph, 1976; Casey, 2009). Emphasize subjective place experience, deep emotional ties, and individually constructed place meaning (Tuan, 1974, 1977, 1980; Relph, 1976) often created through lived experience (Seamon, 2014).
	Place as a locus of attachment (focus on place attachment)	*Top-down information processing/deductive*. We use contextual information as “inputs” [in the form of cognitions, beliefs, attitudes, or other mental representations about a place (Williams, 2014a)] in order to create meaningful, mental perceptions (“output”) (Bernstein, 2010).	*Computational level*. What is the goal of the computation? What problems does the system solve or overcome? (Marr, 1982; David et al., 2004). For example, what is the goal behind forming certain attitudes or beliefs about place?	*Algorithmic-representation level*. What representations does the system use and what processes are employed to manipulate inputs and outputs (Marr, 1982; Bechtel, 2008). For example, what is the dimensionality of place attachment (Kyle et al., 2004; Lewicka, 2005, 2010; Raymond et al., 2010) and how do these dimensions affect behavior (Stedman, 2003; Raymond et al., 2011)?	*Implementation level*. How is the system physically realized in the human brain (David et al., 2004)? For example, how is place attachment realized in the human brain? We are not aware of any place attachment studies in this area of cognitive neuroscience.	
Affordance scholarship (privileges the “fast”)	Place as a perception-action process (focus on affordances)	*Bottom-up information processing*. Perception starts at the stimulus. The real world provides sufficient contextual information for our visual systems to directly perceive what is there, unmediated by the influence of higher cognitive processes (Gibson, 1979).	*Individual perspective*. Affordances exist by virtue of a relationship between the physical properties of the world and the action capabilities of the individual (Gibson, 1979; Jongeneel et al., 2015).	*Social perspective*. For some scholars, a type of affordances exist that are social and that take the form of possibilities for coordinated behavior (e.g., Shockley et al., 2009; Wiltshire et al., 2014). However, it is more commonly assumed that all affordances are “social” because they belong to a shared reality and are bound up with normatively constrained social practices (Kiverstein, 2015).		

Regardless of differences between the above perspectives, they have in common a general emphasis on “slow” progression of meanings and attachment, and are often interested in the social construction of meaning. Unlike in the sense of place scholarship, there is a wider body of literature in Ecological Psychology which “engages the fast,” as reflected in the theory of affordances (**Figure 1**). An affordance refers to the “possibility for action” provided to an individual by an environment – by the substances, surfaces, objects, and other living creatures that surround the social actor (Gibson, 1979). Affordances are the result of real-time or direct perception–action processes in that they depend on the existence of an organism that can perceive them and the actions which the organisms can undertake within a particular setting (Chemero, 2003). For example, a ledge approximately 6 inches high in a public area may function as an edge marker for adults; however, for a young child it can function as a place to sit, as a structure to climb on and to leap over, and as a challenging edge on which to walk (Heft, 2010). The geographic scale of place in this context refers to one’s immediately perceivable environment. Three core principles underpin Gibson’s ecological approach (Chemero, 2003): (1) perception is direct in that it does not involve computational or mental representations; (2) direct perception–action processes are primarily for the guidance of action; and (3) because perception does not involve mental computational or mental representations yet it can still guide behavior, all the information and meaning necessary to guide adaptive behavior must be available in the environment to be perceived (Chemero, 2009).

We now articulate and engage a new branch of place enquiry that engages with the fast as “place as a perception–action process” (**Figure 1**), resulting in the formation of immediately perceived place meanings. This branch takes a bottom-up view of information processing whereby it is assumed that the world provides sufficient contextual information for our visual systems to directly perceive what is there without the need for lengthy cognitive abstraction. It is based on a transactional worldview of psychology, akin to embodied scientific realism, which supports the inseparability of subject and object (Maturana and Varela, 1987; Lakoff and Johnson, 1999). In other words, dynamic, multi-level relationships are possible between elements of mind, body, culture, and environment (Raymond et al., 2017).

In this conceptual paper, we highlight the potential contributions of affordance theory to sense of place scholarship. We first critically discuss a set of “blind spots” in sense of place scholarship and suggest how affordance theory may address them. We then outline a set of future research directions for reconceptualizing sense of place theory to take account of fast and slow processes of cognition associated with perception–action and social construction, respectively.

## Key Blind Spots in Sense of Place Scholarship

### Sense of Place Scholarship Rarely Accounts for Sensory or Immediately Perceived Meanings

Sense of place scholars have largely focused their investigations on the social construction of place attachment or place meaning using interpretative or top-down information processing approaches (**Table 1**). Each approach has a different emphasis on place. Briefly, at the risk of over-simplification, interpretive approaches within “place as a center of meaning” often focus on the meanings that shape actions or everyday experiences (Wagenaar, 2011). Within the diversity of interpretive traditions, sense of place scholars usually employ hermeneutic, discursive, dialogical, and/or phenomenological approaches. In a hermeneutic approach, meaning is generated in the individual subjective mind through the interpretation of texts. Landscape legibility or being able to “read” the landscape is therefore crucial to the formation of a sense of place (Drenthen, 2011). In a discursive approach, place meanings are treated as a social practice that cannot be understood outside of interactional, cultural, and institutional contexts in which they emerge. They are formed through everyday language use and social practice and have important rhetorical relevance (Di Masso et al., 2014). Dialogical approaches emphasize the central role of actions and practices in producing place meaning (West, 2016). A phenomenological approach seeks to qualify the long-term relationship between an individual and a place through lived experience (Relph, 1976; Norberg-Schulz, 1980; Seamon, 2014). Emphasis is placed on subjective place experience, deep emotional ties, and individually constructed place meaning (Tuan, 1974, 1977; Relph, 1976). Meaning is not solely a person–environment relation, but an intersubjective matter of people–environment relations. It is the shared performance of individuals (e.g., by inventing, constructing, and deconstructing structures) that turn lived space into a special place (Graumann, 2002). Much of the early work on sense of place and place attachment focused on this holistic approach (Stedman, 2002).

In contrast, in “place as a locus of attachment” scholars have frequently employed a top-down information processing approach in order to quantify the relationships between activities, physical attributes, and meanings (Moore, 2014). How top-down information processing is used can vary across different levels of processing. In cognitive psychology, three levels of information processing are commonly discussed: computational, algorithmic–representational, and implementational (Marr, 1982; Newell, 1990). Computational level addresses questions such as what is the goal of computation? What problems does the system solve or overcome (Marr, 1982)? Here, we need to consider what drives different attitudes or beliefs about place. In contrast, algorithmic–representational approaches describe how the system represents the problem and what processes are employed to manipulate inputs and outputs (David et al., 2004). Most quantitative research on place attachment has focused on this level of computation. For example, researchers have attempted to psychometrically distinguish between functional goals using the dimension of place dependence and symbolic meanings using the dimension of place identity (Kyle et al., 2004; Hammitt et al., 2006; Raymond et al., 2010; Ramkissoon et al., 2013a; Brown et al., 2015). Psychometric scales that measure these two dimensions of place attachment have been widely used in environmental psychology (Lewicka, 2011), and have been extended to measure the bonds developed between an individual and broader elements of the social and biophysical context to place (Kyle et al., 2005; Jorgensen and Stedman, 2006; Raymond et al., 2010; Ramkissoon et al., 2013b). Implementation-level theory answers the question of how representations and algorithms can be realized physically in the human brain (David et al., 2004). For example, what happens in the human brain when one becomes attached to a given place? No studies have as yet employed neuroscience approaches to the assessment of sense of place.

These differences notwithstanding, common to all these approaches to sense of place is that they rely on a high level of intellectual abstraction of cognitions, beliefs, attitudes, or other mental representations about the physical, social, or personal qualities of a setting and comparatively neglect the role of the local context in direct perception (Vanclay et al., 2008). We also see this intellectual abstraction in studies that question the relative contribution of physical, social, or personal dimensions of place (e.g., Beckley et al., 2007). For example, Williams (2014b) discusses four layers of place meaning: inherent, instrumental, socio-cultural, and identity–expressive (**Figure 1**). Places can have inherent meaning (Lynch, 1960) which transcends any culture, and reflects essential properties of a place that many people perceive. Places can have instrumental meanings associated with their material properties that contribute to desired social or economic goals. They can have socio-cultural meaning which recognizes that places can be socially or symbolically constructed within the cultural, historical, and geographical contexts of day-to-day life (Hay, 1998; Gustafson, 2001; Seamon, 2014). The identity-expressive layer focuses on how individuals become attracted to and attached to place because those places possess intangible emotional, symbolic, and spiritual meaning.

Despite the above, it remains unclear how the *immediately perceived and sensory dimensions* of sight, smell, hearing, taste, and touch (i.e., aspects of sensory experience) contribute to overall place meaning. Crucially, these perceived meanings do *not* involve mental computational or mental representations. Rather, they involve a bottom-up theory of perception whereby perception starts at the sensory input, the stimulus (Bernstein, 2010). Sense of place scholarship has been largely silent on this question with general assumptions made that aspects of the senses represent a particular category of place meaning (Russ et al., 2015). Others imply that sensory experience reflects an intensity of place meaning. For example, Hay (1998) showed that as the amount of time spent in a place increases, the relationship to the place, and in particular the attachment, intensifies and becomes deeper (from “aesthetic experience” to “part of place”). A wider view of the literature suggests that senses themselves are an important element of place perception. Vanclay et al. (2008) suggest that full experience of a place can only be experienced through the senses (smell, taste, feel, sight, and spiritual dimensions), whether we are aware of them or not, and call for the consideration of both intellectual abstraction and sensory perception. They highlight the importance of different senses to people with disabilities, as in the case of the history of “deaf places of silence” which are commonly known to deaf people (Gulliver, 2008). In the marketing and branding literatures, scholars have found that aesthetics and visual cues are powerful ways of tailoring products to the perceived desires of consumers (e.g., Porter, 2013). Other work considers the way in which non-visual senses can, or could, play in the way places are branded (Medway, 2015; Medway et al., 2016).

The complex relations among sensory, inherent, instrumental, socio-cultural, and identity-expressive meanings have not been adequately engaged in the sense of place literature. A holistic assessment would require scholars to accept that sense of place can form through both immediate and direct perception–action processes in addition to the longer-term processes of intellectual abstraction, representation, or computation, which are better represented in the sense of place literature (mainly in terms of social construction). Doing so will require a general willingness to explore sense of place across time, including a consideration of how sense of place forms and changes in response to both immediate perceptions and longer-term processes of social construction.

### Sense of Place Scholarship Pays Little Attention to How Place Meanings Are the Joint Product of Attributes of Environmental Features and the Attributes of the Individual

Many of the debates in sense of place scholarship focus on the relative contribution of social relationships and physical environments to place attachment and place meanings. In psychology, place has been presented as a socio-physical construct comprising of physical, social, and personal/individual components. However, few papers discuss the potential for sense of place to be simultaneously determined by the intersection of attributes of the environmental feature in question and attributes of the particular *individual.* In a review of the literature, Lewicka (2011) found that physical factors have been found to be stronger predictors of place attachment among higher income respondents, whereas social ties are more important among lower income respondents. Scannell and Gifford (2010) found that physical factors were more important reasons for attachment to the city whereas social factors were more important to the home and region. Place meanings can also be influenced by a range of ecological characteristics (Russ et al., 2015) and features of the biophysical environment (Stedman, 2003), whereas others involve pivotal moments or other significant life experiences that happened in a place (Manzo, 2005).

The focus on the relative contribution of different dimensions of place meaning implies a duality between individuals, culture, and the environment in human–nature relationship assessments and stifles discussion on a more holistic concept of “relatedness.” Absent in most discussions is the way in which sense of place is formed based on the nature of the setting, the kind/amount of experience with that setting, and socio-demographic characteristics of the individual (Stedman, 2003). The concept of “situated cognition” is one way to understand such relatedness of the conjoining of people and place. By situated cognition we refer to how meaningful actions are spatially and temporally located (i.e., *situated*) (Chemero, 2009) alongside socially and culturally constructed meaning (Lave and Wenger, 1991; Wenger, 1998). For example, how decisions concerning the speed at which you ride your bike are shaped by your characteristics as an individual (e.g., are you in good physical condition, and are you generally risk averse or risk seeking), the visual perception of a bike trail on a given day (e.g., has it rained and are there muddy spots where you might fall), your previous experiences on that bike trail tied to deeply held place meanings and the social expectations of significant others accrued over time (Raymond et al., 2017). Such dynamic relations imply an inseparability of subject and object (Maturana and Varela, 1987; Lakoff and Johnson, 1999), which sense of place scholarship – at least as currently articulated – cannot clearly explain or describe. Gustafson (2001) has provided one of the most pivotal accounts of the potential for dynamic relations among person, physical environment, and social environment. He found evidence for a network of relational place meanings with some meanings situated in the relationship between self, others, and/or the environment. However, even this work has not seriously engaged the kind of situated cognition we envision.

### Sense of Place Scholarship Implicitly Assumes That the Relationship between Place Attachment and Behavior Is Linear and Not Constituted in Dynamic Relations Among Mind, Culture, and Environment

Scholars have often assumed that sense of place is analytically separable from behavior and therefore it can be used to systematically predict it. For example, results showing that place attachment directly predicts self-reported pro-environmental behavior (Vaske and Kobrin, 2001; Stedman, 2002; Brehm et al., 2013) or place attachment indirectly predicts behavior through values, beliefs, and norms (Raymond et al., 2011). In most instances, the effect sizes of these linear models are only modest (<20%), raising questions about how to model the relationships between sense of place and action, and what the other “missing” predictors of behavior might be. These models also tend to test the relationships between place attachment and behavior at a point in time rather than how these dynamics may vary across time. Rather than a new model, perhaps we require a new set of assumptions about the links between sense of place and behavior. The above assumptions about the relationships between sense of place and behavior change if we engage an alternative worldview of “transactionalism,” which emphasizes the inseparability of subject and object (Maturana and Varela, 1987; Lakoff and Johnson, 1999). Here, dynamic, multi-level relationships are possible between elements of the mind, environmental and cultural system, which imply that one cannot understand aspects of behavior without also understanding aspects of the intertwined socio-cultural system. There is growing evidence for this worldview in human–environment relationship studies, but not in sense of place research *per se*. Cooke et al. (2016) eloquently show that human–environment connections are not produced solely within the mind, but through relations between mind, body, and environment over time. Fischer and Eastwood (2016) find intersections between the co-production of ecosystem structures, ecosystem services, and the social construction of these structures and services. Brown (2016) demonstrates how the experience of textured terrain (e.g., the resistance, gradient, shape lumpiness, and irregularities of the terrain) can produce sensory and emotional experiences that motivate regular exercise.

### Sense of Place Scholarship Does Not Account for How Both Place Meanings and Place Attachment Vary Across the Life Course

The sense of place literature typically engages experience and time over a longer time horizon. It is generally accepted across all approaches that place attachment slowly changes in intensity with one’s length of residence in a given place (Hammitt et al., 2004; Brown and Raymond, 2007), and is shaped by economic, social, political, and other external disruptions to a place (Brown and Perkins, 1992; Feldman, 1996; Devine-Wright, 2009). Research has also considered how place attachment develops across long-term residents, with sequential stages in the development found across time in a given place [Hay, 1998; but see Stedman (2006) for a dissenting view; Rowles, 1983], and the role of nostalgia in facilitating attachments to place (Lewicka, 2011). Scholars have used identity theories to describe how physical changes (both actual and proposed changes) to place may threaten place-based identities (Proshansky et al., 1983; Stedman et al., 2002; Devine-Wright, 2009); and how place attachment may be both threatened *and/or* enhanced across place change (Devine-Wright and Howes, 2010). Place attachment can also vary across life-place trajectories of: long-term residence in a single place, return to the home place, residential mobility with continuity in settlement, residential mobility with discontinuity in settlement, and high residential mobility (Bailey et al., 2016).

Despite the above, the role of place experience in shaping place meanings and attachments across the life course has not been thoroughly considered. Scholars have made a general assumption that place meanings are sustained by regular environmental actions and routines, that are in turn maintained and strengthened across one’s depth of experience with place (Seamon, 1979; Fullilove, 2004). For example, from a phenomenological perspective, place meanings are embedded in stories and metaphors, each highly dependent on context, and embedded or contained within an evolving set of experiences (Patterson, 1998). But how may these meanings change across place experiences at different life stages, and what role do these changing meanings have on one’s overall place attachment? Sense of place scholarship cannot fully answer such questions. They are important to consider because wider psychology research has shown that the connections that we have with family and friends and the experiences we have in place during important transition times in life (e.g., children approaching decisions to leave home) are related to mobility preferences (Elder et al., 1996). Studying these relationships may help us better understand and predict the determinants of mobility and migration, which is a mega-trend of the 21st century and also addresses a major gap in sense of place theory concerning the interrelationships between place meaning and place attachment. Current discussions often become confounded by conflicting views about the relative merits of qualitative vs. quantitative studies implemented over short time spans as opposed to understanding how they together may inform sense of place over different life stages.

## How Affordance Theory Can Address Blind Spots in Sense of Place Scholarship

### Affordance Theory Demonstrates How Direct Perception and Actualization Inform Place Meanings

Two processes underpin the theory of affordances, namely *direct perception* and *actualization* (Kyttä, 2002, 2004). In contrast to most sense of place scholarship, an individual does not require mental computation or representations because the perception of meaningful behaviors is readily available in the environment, hence *direct*. An individual directly perceives what are his/her opportunities for action in an environment given the relations among the observer’s knowledge, intentions, action abilities (constrained by body morphology, physiology, and emotional/intellectual development), and the properties of the environment itself (Canal-Bruland and van der Kamp, 2015). Perception is directly functional for the guidance of action rather than for gathering information (Chemero, 2009, p. 18). *Actualization* is then the processes of complementing environmental opportunities with personal abilities. In other words, the environment provides something that the individual perceives as offering the potential for activity, but actualization of the activity only emerges when the different characteristics of the individual, such as his or her physical abilities, social needs, and personal intentions, are matched in meaningful relations with the environmental features (Kyttä, 2004).

These relations mean that affordances can rapidly change from potential to perceived, used or shaped depending on the relationships between the individual, culture, and setting (Kyttä, 2003). Kyttä (2002) describes this dynamic. All environments have countless numbers of *potential affordances* that no agent has yet perceived. The array of potential affordances available to any given individual is defined by the individual’s qualities such as the children’s physical skills or bodily proportions. The qualities of the individual as well as his or her current intentions and other cultural factors determine which affordances out of all potential affordances the individual perceives in different situations (i.e., *perceived affordances*). Some of these affordances are used in the here and now. Individual and socio-cultural factors can have an influence on what affordances are utilized and when this occurs. It is also possible to actively shape the environment to create new affordances, or to change existing ones, in what is referred to as *shaped affordances*. Modifying the physical environment can open space for the identification of new affordances and new possible activities (Kyttä, 2002, 2004).

Hence, through direct perception and action we create various forms of perceived place meanings related to functional, social, or symbolic elements of a given area. Meanings are assigned to places within one’s immediately perceivable environment. These places have clear material and perceptual components. Hereafter, we refer to them as “immediately perceived place meanings” to distinguish them from the more commonly known place meanings formed through social construction.

### Affordance Theory Demonstrates That Cognitions Are Situated in Relation to the Environment, the Individual, and One’s Socio-Cultural Context

In affordance theory, perception–action process and associated cognitions always occur in a *situation*. Because perception is direct, meaningful actions are always spatially and temporally located (i.e., *situated*), providing information about “here,” “there,” “me,” and “now” (Chemero, 2009). Information perceivable in any given situation will specify patterns of relations between the organism and the environment (Shapiro, 2011). Situations can then have motivating qualities: as in the case of a low ledge for children (as a “climbing place”), or repelling like an aggressive dog (Heft, 2010) suggesting an affective dimension to affordances (Kyttä, 2003; Roe and Aspinall, 2011; Withagen et al., 2012). Thus, patterns at any given situation not only offer the opportunity to act, but also can invite and attract the action (Withagen et al., 2012) or repel and detract the action. Any given place can therefore have an array of positive and negative affordances that promote or inhibit action, respectively.

These perceptions and actions are not only situated with reference to a physical context (Heft, 2013). Associations between bodily experiences and abstract concepts are situated in a socio-cultural context, informed by cultural imperatives, values, and habits (Gibson, 1979; Varela et al., 1991; Leung et al., 2011), and social learning (Bandura, 1977), which can be readily applied to gaining competence in place. Individual and sociocultural factors together determine which of the perceived affordances become *used affordances* within a given experience (e.g., sat on, swam in, climbed on), as in the case of socialization during childhood development. During development, a child learns to perceive not only the affordances for the self, but also how those same objects furnish similar affordances to another (Gibson, 1979). Parents or significant others can introduce children to the conventional meaning of an object by manipulating which objects command attention and demonstrating how to use the object through performing its central function. For example, in a study of a kindergarten in central Norway, some 3–5-year olds needed to be socially invited into physical play, to realize the *potential affordances* for physical activity (Bjørgen, 2016). Encouraging invitations from others, responses, imitations, and sharing moment of fun were of significance in the involvement and duration level of physical activity. Hence, the physical activity is not always created by the children, but requires invitations from others in the environment where they are playing and regulation by significant others who promote certain behaviors.

In summary, this section highlights that immediately perceived place meanings are both temporally and physically located and are influenced by a range of physical and social elements in one’s immediately perceivable environment and by socio-cultural processes.

### Affordance Theory Suggests That Bodily Action Is Constituted within Dynamic Relations among Mind, Body, Culture, and the Environment

Affordance theory suggests that cognition is not an activity of the mind alone, but is instead distributed across the entire relationship situation, including mind, body, culture, and physical environment (Heft, 1989). Through direct perception–action processes an individual actualizes those relations among environment, culture, body, and mind that reflect and support his or her capabilities and intended actions at any moment in time (Kyttä, 2002). How they are actualized depends on the *real-time relationship* between a mental system in a body with particular capabilities with an environment that offers opportunities for acting on those services. Hence, bodily action is constituted within dynamic relations.

The coupling of perception and action in the social affordance literature is one example of these dynamic relations. It has been found that eye gaze patterns influence postural coordination, and gaze co-ordination is related to mutual understanding [see Shockley et al. (2009) for a review]. For example, pairs of individuals who are asked to perform a rhythmic task such as rocking in a chair rock independently in their chairs are pulled to spontaneously synchronize their movements (Richardson et al., 2005, 2007). The patterns of behavior that occur between the two individuals rocking independently in separate chairs with no mechanical (only informational) links obey the same dynamics as coupled components (Marsh et al., 2009). Studies have also shown coupling across a range of other behaviors including walking, running, and plank lifting (Anderson et al., 2012).

It is important to note that these perception–action processes are not static, but since they are related to activities, they happen over time and their actualization changes the subsequent patterns of relations between individual and environment (Chemero, 2009). In other words, an individual’s perception–action in the environment influences both surrounding (e.g., by manipulating objects, affecting others, moving) and abilities (e.g., by learning, acquiring new skills), and in doing so opens up possibilities for new activities and thus novel or reshaped patterns of affordances (Shotter, 1983; Heft, 1996). Raymond et al. (2017) provide the example of a mountain bike rider to show a dynamic relation among the condition of the rider, perception of the environment, and riding speed (behavior). They note that the act of riding improves individual body condition and confidence, which in-turn has an effect on what kind of environment can be perceived as safe and enjoyable. Skills are embodied by repeatedly practicing mountain-biking allowing a rider to perceive a rocky and wet slope as safe and enjoyable, thereby enabling the rider to approach it and enjoy it at faster riding speed.

In summary, this section highlights that elements of our own mind and bodily condition inform what types of meanings we immediately perceive in a given place. Human have the ability to learn new skills and to improve their physical condition which opens the potential for new forms of immediately perceived place meanings. We also change a place through our actions, which opens spaces for new meanings.

## Discussion

The objective of this paper was to highlight the potential contributions of affordance theory to sense of place scholarship. We asserted that most sense of place scholarship is preoccupied with the “slow” in that most research suggests that place meanings as slow to evolve, sometimes not matching material or social reality (lag effects), and thus as tending to inhibit change. Little attention is paid to the role of sensory or immediately perceived meanings in the formation of sense of place; how place meanings are the joint product of attributes of environmental features and the attributes of the individual (i.e., the importance of situated cognition); the non-linear dynamics between sense of place and behavior, including the dynamic relations among mind, culture, and environment; and how place meanings vary across the life course. In contrast, affordance theory engages the “fast” – the more immediate perception–action processes between an individual and their social and cultural environment. Affordance theory demonstrates how direct perception and actualization inform immediately perceived place meanings that cognitions are situated in relation to the environment, the individual, and one’s socio-cultural context, and behavior is constituted within (and simultaneously determined by) dynamic relations among mind, body, culture, and the environment.

How do these different views on human–environment relationships influence our approach to sense of place research? Rather than viewing sense of place as exclusively (or even primarily) a social construction or representation, we suggest it could be seen as a *property of the relationship between perception–action and social construction processes both within and across place-based experiences*. Along these lines, we encourage sense of place researchers to assess the relationships among immediately perceived place meanings and other forms of place meaning that are socially constructed through longer-term processes of cognition and how each may contribute – independently, and collectively – to one’s place attachment. To understand these relationships, we encourage place scholars to move away from a focus on concept development and measurement, based firmly in the social construction approach, to enquiries of place as a *multi-channel process* which provides for an understanding of the relational dynamics between perception–action processes and socially constructed processes. Integrating the perceptual and conceptual domains will require place scholars to engage with the theory of embodied cognition, dynamic systems, as well as multiple views of cognition and behavior. In the next section, we propose pathways through which to consider the potential relationships between these two understandings of human–environment relationships.

### Embracing the “Fast” and “Slow” in Future Sense of Place Research

Future research could investigate how the qualities of both sense of place scholarship and affordance theory could be applied to solve important issues in sense of place scholarship, such as how place attachment may form and change in a given place. The dual-process theory of higher cognition could be a fertile ground through which to explore or examine the intersections between place meanings formed through socially constructed process and meanings formed through affordances, i.e., perception–action process. According to this theory, both slow and fast forms of cognition exist (Kahneman, 2003; Evans, 2010; Evans and Stanovich, 2013). Type 1 cognition is grounded in perception and intuition – thinking is fast, automatic, effortless, and associative, while Type 2 which is grounded in reasoning – thinking is slow, serial, controlled, effortful, and rule-governed. Type 1 generates impressions of the attributes of objects of perception and thought. In contrast, Type 2 is involved in judgments, irrespective of whether they originate in impressions or in deliberative reasoning (Kahneman, 2003). While there have been a number of criticisms of dual-process theories (see overview in Evans and Stanovich, 2013), there is general acceptance that Type 1 represents a set of modes of cognition associated with rapid autonomous processes that yield habitual responses unless they are intervened on by higher order reasoning processes of Type 2 (Evans and Stanovich, 2013). Within each type there are modes of cognitive processing styles or thinking dispositions (Stanovich, 2009) which can vary continuously according to personality characteristics and cultural factors (Evans and Stanovich, 2013).

Following this view, is it possible that direct perception–action processes operate as a subset of Type 1, fast, automatic processes (following Herschbach, 2015)? In contrast, can socially constructed process be considered a subset of Type 2, slow processes? Three research areas worth considering under this line of thought are:

#### Research Direction 1 – Investigate How Sense of Place Is Both Directly Perceived (Type 1) and Socially Constructed in a Given Place (Type 2)

Perceived meanings, as we have described above, may play a bigger role in “sense of place” than we typically think. We propose that in any experience in life, sense of place can be associated with immediately perceived place meanings (related to Type 1 cognition) and/or place meanings formed through longer-term processes of social construction (related to Type 2 cognition). For example, the fireplace a couple sits next to after getting married enables the used affordance of warmth and light, plus a cozy atmosphere. Equally, that fireplace can be related to a diverse set of inherent, instrumental, socio-cultural, and identity–expressive meanings (Williams, 2014b), which can be positive and negative in nature (Manzo, 2005). At the time of the wedding they may be related to feelings of romance and love, but if the fireplace was the backdrop for the scene of agreement to divorce it could also be associated with feelings of grief and loss, or if during a winter power outage, simply a pragmatic source of warmth or cooking. It follows from above that direct perceptions can be repeated in behavior.

We also propose that the relationships between direct perceptions and social construction are unlikely to be direct and linear. At small geographic scales, we hypothesize that place attachment will be a property of both immediately perceived and socially constructed place meanings, and that these meanings collectively help guide behavior. To investigate this hypothesis, a fruitful area of enquiry may be to specify physical characteristics of the environment that are perceived positively for a certain set of affordances and are known (at least anecdotally) to be a source of place attachment. Starting with a set of known relationships would help researchers to identify the mechanisms through which Type 1 and Type 2 processes occur.

Understanding the duration of focus of direct perception and social construction is also crucial to this line of enquiry. It is assumed that direct perception is immediate and social construction takes longer, but what are the actual time differences? Quasi-experiments may play an important role in understanding the time differences in cognitions resulting from direct perception and social construction so as to form a more holistic understanding of place.

To date we have avoided the question of how different types of place meanings inform each other at different geographic scales. To understand how sense of place is both perceived and socially constructed, we need to clarify the spatial scale of “place” and thus to what extent an affordance is a “place.” Here, we argued that there are parallels between affordances and sense of place when focusing on one’s immediate perceivable environment, such as a room, home, street, or small urban park (i.e., fine geographic scales). Yet how do direct perception and social construction occur at coarser geographic scales such as a neighborhood, city, or region? In short, we don’t yet know. An important future research direction is to examine whether affordances can be nested or sequenced in ways that enable us to understand direct perception at larger spatial scales. Such research would need to challenge some of the fundamental assumptions of affordance theory concerning how humans directly perceive the environment. For example, researchers would need to consider the potential for a collection of affordances in a place, a network of places in the neighborhood, a set of familiar and unfamiliar neighborhoods in a metropolitan area, etc. We consider this question of the scalability of affordance-based thinking an exciting area of future inquiry.

#### Research Direction 2 – Explore and Examine Whether Perceived (Type 1) and Socially Constructed (Type 2) Dimensions of Place Relate to One Another When Perceived Meanings Become Unsituated

The previous research direction investigates whether place as a perception–action process and place as a socially constructed process exist concurrently in a given place. But how do they relate? We propose that perceived and socially constructed meanings relate when perceived meanings become unsituated. This may occur through the process of “offline cognition.” According to Wilson (2002), humans build up in long-term memory a set of reactions, movements, sensations, perceptions, feelings which are available for various purposes including cultural ones. In this process, mental structures that originally evolved for perception or action are co-opted and run “off-line,” to assist in thinking and knowing (Glenberg, 1997; Wilson, 2002). Just thinking about an object produces states as if the object were actually there, as does perceiving a symbol, such as the name of the person or object (Spackman and Yanchar, 2014). Cognition can therefore become “unsituated.” The evidence for offline cognition is widespread. It is included in discussions of mental imagery (including visual, audio, and kinesthetic imagery), working memory, episodic memory, implicit memory, and reasoning and problem-solving [see Wilson (2002) for a review].

No empirical work has tested the potential relationships between perceived and socially constructed meanings using ideas of offline cognition, but we can describe it from a practical perspective at fine geographic scales such as a room. Imagine a room in our home that we have been living in for a long time. We enter and exit that room multiple times on a daily basis and our action is guided by a range of perceptions concerning the size, light, and warmth of that room. Now imagine that we get married in that room. Through processes of social construction, that room is now associated with a range of socio-cultural meanings like love and social connection, and inherent meanings like the ambience of the room on the wedding day. Through processes of offline cognition, these perceived meanings associated with the sights, smells, and sounds of the wedding day become engrained in memory and in language, which provides the pathway through which perceived and socially constructed meanings relate.

We propose that perceived and socially constructed meanings are most likely to conjoin when memories are activated. In future studies, researchers could draw on phenomenological approaches in order to investigate the relationships between different components of living memory. That is, how different types of affordances are contained within (or independent to) inherent, instrumental, socio-cultural, and identity–expressive meanings within a given place and/or across places. Also, researchers could identify which types of affordances become “lost in space,” i.e., are rarely associated with different types of socially constructed meanings, but rather are part of our everyday navigation through life.

#### Research Direction 3 – Explore How Place Attachment Changes Over Different Stages of the Life Course Based upon Dynamic Relationships between Processes of Social Construction and Perception–Action

While research has considered how place attachment varies with respect to length of residence, place disruption, or nostalgia, no studies have considered how the structure of place attachment may change with reference to different forms of place experiences and place meanings across the life course. By bringing together the qualities of sense of place and affordance theories, we propose that *place attachment could be considered as an emergent property of the dynamic sets of meanings associated experiences across the life course*. By this we mean that types of perceived and socially constructed meanings formed during experiences at an early life stage (e.g., during a 6th birthday) are likely to inform the perceived and socially constructed meanings during experiences at a later life stage (e.g., getting married), assuming those experiences occur in the same place. The integration of the affordance perspective and how it relates – for example – to the changes in physical capacities associated with aging (e.g., the ledge we referred to earlier may be a challenging climbing place to a child, but too easy for the adult, and beyond the capacity of the elderly) may prove a very useful framework for understanding changes in attachment through the life course (e.g., Cuba and Hummon, 1993) or among the elderly in particular (Rubinstein and Parmelee, 1992).

We don’t know how these immediately perceived and socially constructed place meanings combine within and across time to inform changes in the intensity or structure of place attachment. To establish this process, we require new mechanisms for not only examining how place as a perception–action process and socially constructed process relate at any given point in time (i.e., research area 2), but also how they may relate across significant experiences in a given place. Longitudinal research is needed to establish whether and how the memories associated with given affordances earlier in life inform later experiences in the *same place*.

Certain elements of early place experiences could also be transferrable to new settings, requiring an improved understanding of how perception–action processes and socially constructed meanings relate across time and *different places*. Longitudinal research is also needed to understand how the affordances perceived in one place early in life are held in memories to inform the affordances and social constructions of other places later in life.

Understanding place attachment as an emergent property of a complex system also has major implications for the assessment of the relationships between place attachment and pro-environmental behavior. If place attachment is an emergent property of both perception–action and socially constructed processes which are changing across one’s time in place then such phenomena cannot be fully understood using traditional linear cause and effect models, as often applied in place research when understanding place as a locus of attachment. Rather, we encourage a new approach to the assessment of pro-environmental behavior which is based on non-linear, complex dynamics. Such dynamics are already the source of intense investigation through areas such as social learning (Cheng and Mattor, 2010), complexity science, and sustainability transitions (Andersson, 2014) and co-production of knowledge (Armitage et al., 2011; Tengö et al., 2014; Reyers et al., 2015), and initial thinking has occurred in the sense of place realm as well (Stedman, 2016; Masterson et al., 2017). We urge a more spatial and relational view of these dynamics recognizing how individual minds and bodies and constituted within places that are both perceived and socially constructed, and how both direct perception and abstractions can lead to formation and change in behavior across places and time.

### Implications of Fast and Slow Processes for the Design and Management of Urban Landscapes

If urban settings are repositories for a range of socially constructed and perceived meanings (Research Direction 1) then these settings need to be designed with both fast and slow cognitive processes in mind and thus multiple layers of place meaning. By layers we mean planning, designing, and implementing new forms of architecture in urban landscapes that cater for clusters of different types of immediately perceived and socially constructed place meanings, including functional, affective, and symbolic. These clusters could be tailored to different user groups so as to address important elements of environmental justice (Raymond et al., 2016).

Stemming from Research Direction 2, it may be possible for urban environments to be designed with affordances that immediately evoke different forms of place meanings. Such urban designs may have multiple important uses for highly mobile individuals or migrants seeking to integrate into new communities rapidly. Certain types of affordances could be created in urban environments to bridge place meanings between their place of origin and their new (sometimes temporary) place of residence. For example, by creating “open spaces” which enable new migrants to shape and reshape affordances in that setting through art, craft, and music, informed by place meanings and memories from their place of origin. However, we acknowledge that designing urban environments for a diversity of meanings can lead to the potential for conflicts between different interest groups, which also needs to be managed (Stedman, 1999).

Accepting place attachment as an emergent property (Research Direction 3) requires urban planners to take account of both short-term and long-term processes of cognition when designing cities. For example, forecasting how citizens’ experiences in a given setting could inform future experiences (and associated immediately perceived and socially constructed meanings), as opposed to designing solely for immediate, functional uses. This strategy would also suggest that city planners recognize that the end goal may not be to “increase place attachment” *per se*, but rather create settings that cater for a diversity of experiences and place meanings, each informing diverse (and sometimes oscillating) trajectories of place attachment across time. It also requires planners to recognize that initiatives for encouraging “sense of place” among different ethnic and migrant groups may achieve fast results in some areas (e.g., providing a playground so that children have the opportunity to play in their local environment), but have slow results in other areas (e.g., building the social capital so that individuals feel they belong in that place).

## Conclusion

In this paper, we urged a systematic consideration of how both slow and fast processes of cognition inform sense of place scholarship. We asserted that sense of place scholarship has been conservative, non-dynamic, and principally focused on aspects of place meaning that unfold over time through a process of social construction. Theory development has largely excluded the role of immediate sensory and direct perception–action processes in meaning making, otherwise referred to as immediately perceived place meanings, but instead focused on place meanings formed through longer-term processes of social construction. In response, we suggest how affordance theory could overcome a number of blind spots in sense of place scholarship and then suggest research directions for empirically justifying how place as perception–action processes (a subset of Type 1 thinking in the dual-process model) and place as socially constructed processes (a subset of Type 2 thinking) relate to each other across place experiences and time. Reconceptualizing sense of place as fast *and* slow presents opportunities to consider how immediate perceptual processes can contribute to longer-term processes of social construction and vice versa. It also paves the way to addressing one of the most contentious aspects of sense of place scholarship and wider psychology: how processes of intellectual abstraction and computation based on interactionist worldviews can be united with immediate sensory experience based on transactional worldviews to better account for not only for place meanings and place attachment, but also environmental behavior across the life course.

## Author Contributions

CR reviewed the literature and developed the key arguments for the paper, also wrote most sections of the paper with additions from MK and RS. MK provided insightful contributions to the affordance theory section of the paper, and to the future directions and management implications sections. RS provided insightful contributions regarding the argumentation and framing of the paper, and also provided key theoretical insights into sense of place, and the discussion section of the paper.

## Conflict of Interest Statement

The authors declare that the research was conducted in the absence of any commercial or financial relationships that could be construed as a potential conflict of interest.
